# The complete genomic sequencing of a *Klebsiella pneumoniae* isolated from a patient suffering from urinary tract infection

**DOI:** 10.1128/mra.01254-23

**Published:** 2024-03-21

**Authors:** Lingfang Di, Chunyan Gu, Sayyed Salman, Yafei Li

**Affiliations:** 1 Department of Clinical Laboratory, Tongxiang First People's Hospital, Tongxiang, Zhejiang, China; 2 Department of Hematology, Tongxiang First People's Hospital, Tongxiang, Zhejiang, China; 3 Collaborative Innovation Center for Diagnosis and Treatment of Infectious Diseases, State Key Laboratory for Diagnosis and Treatment of Infectious Diseases, the First Affiliated Hospital, College of Medicine, Zhejiang University, Hangzhou, China; Indiana University, Bloomington, Bloomington, Indiana, USA

**Keywords:** *Klebsiella pneumoniae*, genomic sequencing

## Abstract

The present study examines the genomic sequence of *Klebsiella pneumoniae* strain ACESH02121hy, which possesses a genome size of 5,281,767 bp. The strain was obtained from a patient’s urine sample presenting symptoms associated with urinary tract infection.

## ANNOUNCEMENT

This study analyzes the presence of *Klebsiella pneumoniae* in a patient diagnosed with urinary tract infection. Urine sample from the patient was cultured on a 5% blood agar plate and incubated at 37°C overnight. Bacterial strains were identified by selecting and streaking colonies with distinct morphologies on MacConkey agar to obtain pure cultures. Following this, matrix-assisted laser desorption/ionization time-of-flight mass spectrometry (Bruker, Bremen, Germany) was used for their definitive identification (1). After identification, pure culture was stored in microbank tubes (glycerol 20% solution) at −80°C for further analysis.

The DNA was extracted using the SteadyPure universal genomic DNA extraction kit (AG21010) from a culture diluted to an optical density of 2–5. The culture was derived from previously stored frozen stock and was incubated overnight at 37°C on Mueller Hinton agar. NanoDrop 2000 UV-visible (UV-Vis) spectrophotometer (Thermo Scientific, Waltham, MA, USA) and Qubit (version 2.0) fluorometer (Thermo Scientific) were used to evaluate the quality and amount of the extracted DNA (1) and then processed for both Oxford Nanopore and Illumina sequencing. For the short reads, DNA was fragmented to a size of 350 bp through sonication (Diagenode Picoruptor) using a sonication period of 15 seconds and six cycles. Then, Nextera XT kit (Illumina, USA) was used to prepare the sequencing library, and the sequencing was completed on the Illumina NovaSeq 6000 platform. After ligating the A-tailed fragments with paired-end adaptors using PE150 strategy, they were amplified with PCR using 500-bp insert. The PCR products were then purified through the AMPure XP system (USA), and the quality of the library was evaluated using the Agilent 5400 system (USA) and quantified through QPCR (1.5 nM). Fastp (version 0.23.1) was used to analyze short-read sequencing quality by discarding paired reads with adapter contamination, followed by over 10% unknown bases or more than 50% low-quality bases (Phred quality <5) at the machine’s default parameters except where otherwise noted. The DNA library was generated using the ligation sequencing kit (SQK-LSK114) without sheering the DNA for the long reads. Subsequently, raw reads from the PromethION were initially assessed using the MinKNOW (version 23.07.12) software for real-time QC metrics and were processed with the Oxford Nanopore Technologies Guppy software (version 0.17.1) for the base calling. The Nanofilt tool (version 2.8.0) was used to filter the reads. Any readings with a mean *Q*-score below 10 were eliminated from subsequent analysis.

While Unicycler (version 0.4.7) was used for the hybrid assembly (2), the sequence was then submitted to National Center for Biotechnology Information (NCBI) for annotation and bioinformatics analysis by NCBI Prokaryotic Genome Annotation Pipeline (version 6.6) (3, 4). It was performed using a best-placed reference protein set, and acquired antibiotic resistance genes were identified by ResFinder (version 4.1). Related metrics are listed in Table 1. The complete genome size of *K. pneumoniae* ACESH02121hy is 5,281,767 bp. The GC content was approximately 56.6%, with an L50 value of 1. Antimicrobial resistance gene analysis showed that the chromosome contained two antimicrobial resistance genes, *fosA* (fosfomycin) *oqxA* and *oqxB* (quinolone) *bla*_SHV-101_ (β-lactam). The PlasmidFinder analyses showed that *K. pneumoniae* has three plasmids: the first plasmid has 13 antibiotic resistance genes, along with the *bla*_TEM-1B_ and *bla*_CTX-M-3_ (β-lactam) genes; the second plasmid carrying *bla*_NDM-5_ (β-lactam) and the third plasmid have no antibiotic resistance gene.

**TABLE 1:** Features of the complete whole-genome sequences of *Klebsiella pneumoniae* strain ACESH02121hy

Feature		
Long read	Number of reads	95,373.0
	Total bases	1,091,445,449.0
	Mean read length (bp)	11,444.0
	Mean read quality	13.6
	Median read length (bp)	10,257.0
	Median read quality	14.1
	N50 read length (bp)	12,342.0
Short read	Total bases	11,554,920
	Raw base (Mb)	1,735
	Raw read 1 Q20 (%)	98.02
	Raw read 2 Q20 (%)	98.18
	Clean base (Mb)	1,733
	Clean read 1 Q20 (%)	98.03
	Clean read 2 Q20 (%)	98.19
	GC (%)	56.96
Assembly	Genome size (bp)	5,281,767
	Long read coverage (*X*)	400
	Short read coverage (*X*)	340
	GC content (%)	57.4
	N50 value	5,154,670
	L50 value	1
Plasmid1	Sequence size (bp)	75,617
	GC content (%)	52.2
Plasmid2	Sequence size (bp)	49,020
	GC content (%)	46.9
Plasmid3	Sequence size (bp)	2,460
	GC content (%)	48.9
	Resistance gene (antimicrobial)	
	Chromosome	*fosA* (fosfomycin)
		*OqxA* and *OqxB* (quinolone)
		*blaSHV-101* (β-lactam)
	Plasmid1	*aac(6')-Ib-cr* and *aadA16* (Aminoglycoside)
		*tet(A)* (tetracycline)
		*mph(A)* (macrolide)
		*qnrS1* and *qnrB91* (quinolone)
		*ARR-3* (rifamycin)
		*dfrA27* and *sul1* (folate pathway antagonist)
		*bla*_TEM-1B_ and *bla*_CTX-M-3_ (β-lactam)
		*qacE* (quaternary ammonium compound)
		*floR* (amphenicol)
	Plasmid2	*bla*_NDM-5_ (β-lactam)

## Data Availability

The whole-genome shotgun sequencing data for *Klebsiella pneumoniae* strain L2121hy has been submitted to the DDBJ/ENA/GenBank repositories, bearing the accession number JAXASD000000000. The version of these data referenced in the article is version AXASD010000000. Associated with this version are BioProject accession number PRJNA1042619 and BioSample accession number SAMN38322493, along with Oxford Nanopore Technologies SRA number SRR26882527 and the Illumina SRA number SRR27292845. It is important to note that the version described in this article is the initial release.
